# The relationship between headache-attributed disability and lost productivity: 2. Empirical evidence from population-based studies in nine disparate countries

**DOI:** 10.1186/s10194-021-01362-z

**Published:** 2021-12-18

**Authors:** Hallie Thomas, Simple Futarmal Kothari, Andreas Husøy, Rigmor Højland Jensen, Zaza Katsarava, Michela Tinelli, Timothy J. Steiner

**Affiliations:** 1 Department of Neuromedicine and Movement Science, NTNU Norwegian University of Science and Technology, Edvard Griegs gate, Trondheim, Norway; 2 Department of Dentistry and Oral Health, Section of Orofacial Pain and Jaw Function, Aarhus University, Aarhus, Denmark; 3 Hammel Neurorehabilitation Centre and University Research Clinic, Hammel, Denmark; 4 Danish Headache Centre, Department of Neurology, University of Copenhagen, Rigshospitalet Glostrup, Glostrup, Denmark; 5 Evangelical Hospital Unna, Unna, Germany; 6 Department of Neurology, University of Duisburg-Essen, Essen, Germany; 7 EVEX Medical Corporation, Tbilisi, Georgia; 8 IM Sechenov First Moscow State Medical University (Sechenov University), Moscow, Russian Federation; 9 Care Policy Evaluation Centre, The London School of Economics and Political Science, London, UK; 10 Division of Brain Sciences, Imperial College London, London, UK

**Keywords:** Headache disorders, Disability, Impairment, Lost productivity, Association analysis, Health economics, Health policy, Global campaign against headache

## Abstract

**Background:**

Headache disorders are disabling, with major consequences for productivity, yet the literature is silent on the relationship between headache-attributed disability and lost productivity, often erroneously regarding the two as synonymous. We evaluated the relationship empirically, having earlier found that investment in structured headache services would be cost saving, not merely cost-effective, if reductions in headache-attributed disability led to > 20% pro rata recovery of lost productivity.

**Methods:**

We used individual participant data from Global Campaign population-based studies conducted in China, Ethiopia, India, Nepal, Pakistan and Russia, and from Eurolight in Lithuania, Luxembourg and Spain. We assessed relationships in migraine and probable medication-overuse headache (pMOH), the most disabling common headache disorders. Available symptom data included headache frequency, usual duration and usual intensity. We used frequency and duration to estimate proportion of time in ictal state (pTIS). *Disability*, in the sense used by the Global Burden of Disease study, was measured as the product of pTIS and disability weight for the ictal state. *Impairment* was measured as pTIS * intensity. *Lost productivity* was measured as lost days (absence or < 50% productivity) from paid work and corresponding losses from household work over the preceding 3 months. We used Spearman correlation and linear regression analyses.

**Results:**

For migraine, in a linear model, we found positive associations with lost paid worktime, significant (*p* < 0.05) in many countries and highly significant (*p* < 0.001) in some despite low values of R^2^ (0–0.16) due to high variance. With lost household worktime and total lost productivity (paid + household), associations were highly significant in almost all countries, although still with low R^2^ (0.04–0.22). Applying the regression equations for each country to the population mean migraine-attributed disability, we found pro rata recoveries of lost productivity in the range 16–56% (> 20% in all countries but Pakistan). Analysing impairment rather than disability increased variability. For pMOH, with smaller numbers, associations were generally weaker, occasionally negative and mostly not significant.

**Conclusion:**

Relief of disability through effective treatment of migraine is expected, in most countries, to recover > 20% pro rata of lost productivity, above the threshold for investment in structured headache services to be cost saving.

## Background

Headache disorders are the cause of disabling ill health, awareness of which has increased dramatically over the last decade [1–10]. Among the consequences is lost productivity: there is clear workforce-based evidence of this from a car-manufacturing company in Turkey [11] and population-based evidence of national losses, to the detriment of gross domestic products, in, for example, China [12], India [13], Nepal [14], Ethiopia [15] and Zambia [16].

In an earlier paper, we searched the literature for evidence of the relationship between headache-attributed disability and lost productivity, recognising the importance of this in the contexts of health care and policy [17]. The crucial question was, and still is: “To what extent might alleviation of the symptom burdens of headache disorders – the principal cause of disability – be expected to reduce the lost-productivity burdens?” Economic analyses of care delivery, for example by structured headache services [18], hinge on the answer if they are to include indirect costs – the larger part by a substantial margin of all costs [12–16, 19–24]. We found the literature not only silent on the relationship and offering no response to the question but also confused, attaching multiple and often erroneous meanings to the term “disability” [17]. We concluded that the answer must come from empirical studies, that the need for these was high and that clear definitions of terms were a prerequisite [17].

Our aim here, therefore, was to evaluate the relationship between headache-attributed disability on the one hand and lost productivity on the other using empirical data. We focused on migraine and medication-overuse headache (MOH), the two most disabling common headache disorders. We had access to individual-participant data (IPD) from a number of population-based surveys conducted or supported by *Lifting The Burden* (LTB), the UK-registered non-governmental organisation conducting the Global Campaign against Headache [25–27] in official relations with the World Health Organization [28]. These data included symptom burden, allowing calculation of impairment, disability (in the sense used within the Global Burden of Disease (GBD) studies [2–8, 17]), and lost productive time from paid work and household chores.

## Methods

### Definitions

For simplicity, and because of lack of data, we defined symptom burden only in terms of headache episodes (ictal burden), characterised by frequency, duration and headache intensity. We ignored burden arising from associated symptoms such as nausea, and interictal burden [29], both usually of lesser importance.

We defined headache-attributed *disability* as in the Global Burden of Disease (GBD) studies, expressed at population level in years lived with (or lost to) disability (YLDs) [2–8, 17]. While this did not align with general understanding of the term (impact of impairment on a person’s functional ability), it suited our purpose well because it reflected ill health in a much broader sense [17, 30, 31]. Furthermore, it was amenable to expression in units suited to economic evaluations.

We defined headache-attributed *lost productivity* in terms of absenteeism from or reduced productivity in paid work and corresponding losses in household work, ignoring, again for simplicity, the economically less important detriment to social participation.

### Data acquisition

We used IPD from LTB’s population-based studies in six disparate countries with large sample sizes (> 2000): China [32], Ethiopia [15], India [33], Nepal [14], Pakistan [34] and Russia [35]. We also used IPD from selected populations, in Lithuania, Luxembourg and Spain, surveyed in the Eurolight project [36, 37].

### Ethics

Ethics approvals in all countries had been obtained from the respective national and/or local ethics committees of the countries or areas included; these are reported in the respective publications [14, 15, 32–37]. In line with these approvals, all participants had given informed consent to collection of their data for purposes of assessing headache-attributed burden.

### Sampling and data collection in population-based studies

Data from these studies were collected using standardised methodology [38, 39]. The detailed methods in each country, with any adaptations necessary, are presented elsewhere [15, 32, 36, 40–45].

Each study was a cross-sectional survey employing randomised cluster sampling to reflect the diversities of the country or area, thereby generating representative samples. The enquiry procedure in all countries involved unannounced door-to-door visits at random households (“cold-calling”) within each cluster. One adult member (18–65 years) of each household was randomly selected for interview. All interviews were conducted using the Headache-Attributed Restriction, Disability, Social Handicap and Impaired Participation (HARDSHIP) questionnaire [39], translated into the local language(s) in accordance with LTB’s translation protocols [46]. HARDSHIP included demographic enquiry, a neutral headache screening question followed by diagnostic questions based on the International Classification of Headache Disorders (ICHD) [47], and enquiry into elements of headache-attributed burden including symptom burden and lost productive time (see below) [39].

### Sampling and data collection in Eurolight

The detailed methods of Eurolight have been published elsewhere [36]. The project took the form of surveys, using a structured questionnaire that was a close derivative of HARDSHIP, in ten countries of the European Union. Sampling methods varied between countries. We used IPD only from Lithuania and Luxembourg, with samples reasonably considered to be population-based [36], and from Spain. Two datasets were available from Spain: one gathered from the employees of various companies operating in the national postal services, and the other from members of Asociación Española de Pacientes con Cefalea (AEPAC) and their families [36, 37]. We used both.

### Symptom burden

Symptom enquiry relevant to estimation of disability (as defined) included headache frequency (days/month) and usual attack duration (minutes, hours or days). For usual headache intensity, response options were “not bad”, “quite bad” and “very bad”, which we interpreted as mild, moderate and severe.

### Lost productivity

Enquiry into lost productive time during the preceding 3 months used the Headache-Attributed Lost Time (HALT) questionnaire [48] as a module within HARDSHIP [39]. Two questions (1 and 2) counted days in that period (i) completely missed from paid work (absenteeism) and (ii) with < 50% productivity (less than half achieved of what was normally expected) while at work (presenteeism), in each case because of headache. Two similar questions (3 and 4) asked for days of household work (iii) completely missed and (iv) with < 50% productivity [48].

### Analysis

#### Diagnosis

Diagnoses in all studies, of only the most bothersome headache when a participant reported more than one type of headache, were made by diagnostic algorithm applying modified ICHD criteria [39]. Headache on ≥15 days/month was first identified, and diagnosed as probable MOH (pMOH) when acute medication overuse had also been reported. In all other cases, for the purposes of this analysis, the algorithm first identified definite migraine, then (for exclusion) definite TTH, then probable migraine [38, 47]. Definite and probable migraine were combined for further analyses.

#### Statistics

We used symptom and lost-productivity data attributable to migraine or pMOH. We combined the Eurolight data from Lithuania and Luxembourg for analysis. We summarised the IPD as means with standard errors (SEMs) and/or standard deviations (SDs) as appropriate. We calculated time in ictal state (TIS) at individual level as the product of headache frequency (F) and average duration converted into days (D), and expressed this as a percentage of total days (pTIS). Whereas surveys had collected average attack duration as continuous data in hours, F was reported in headache days/month, not attacks/month. Therefore, as a correction whenever D > 1 (*ie*, average duration > 24 h), we used F rather than F*D, as a conservative approximation, to express TIS. We censored all cases for whom F < 4/year and/or D < 0.04 (< 1 h), and any surviving cases for whom pTIS< 0.5% (equivalent to 4 h/month of symptoms), judging these to be clinically insignificant, not on the spectrum of interest for headache care and/or uninformative about the relationship under enquiry.

We calculated *disability* at individual level, as a percentage, as the product of pTIS and the disability weight (DW) for the ictal state of the disorder in question from GBD2015: 0.441 for migraine and 0.217 for MOH [49].

In separate analyses of migraine, we incorporated reported usual intensity as a multiplier of pTIS in place of DW (a constant), better to reflect symptom burden at individual level as suggested in our earlier paper [17]. We referred to the product of pTIS and usual intensity, calculated at individual level and expressed in arbitrary units, as *impairment*.

We expressed *lost productivity* at individual level in accordance with responses to the four questions from HALT in whole days/3 months, equating, according to accepted methodology, “less than half achieved” to “nothing achieved” and counterbalancing this by equating “more than half achieved” to “everything achieved” [48].

We assessed associations between these: headache-attributed disability or headache-attributed impairment as independent variables and lost productivity as dependent variable for each country and each headache type. We applied Spearman correlation (calculating the coefficient r_s_) and linear regression analyses to the individual measurements of these derived from the IPD, calculating the coefficients β (degree of change in the dependent variable for every unit of change in the independent variable) and R^2^ (the proportion of variance in the dependent variable that can be explained by the variance in the independent variable). For the regression analyses we generated scatter plots to show variability in the IPD, with linear trendlines. To obviate axis compression in these plots, we removed the very small numbers of extreme outliers (≤4 per plot) by visual inspection.

We used Statistical Package for the Social Sciences (SPSS), version 26.0 and Excel Professional Plus. We considered *p* < 0.05 to be significant and *p* < 0.001 to be highly significant.

## Results

Although the association analyses were performed on the IPD, these are summarised for interest by headache type and country in Table 1 (pTIS, headache-attributed disability and headache-attributed impairment) and Tables 2 and 3 (lost productive times). The data available to us from China did not include medication use in those with headache on ≥15 days/month, so we could not identify pMOH. Table 1 shows mean pTIS for migraine varying two-fold between countries in the range 4.5–9.4%, with medians, always considerably lower than means, indicating high degrees of skewedness. Disability and impairment varied accordingly. For pMOH, mean pTIS varied from 22.7% to 61.6%. Tables 2 and 3 likewise show high variability between countries in lost productive time, in total between 4.4 and 14.0 days/3 months for migraine and between 14.0 and 34.7 days/3 months for pMOH. It is worth noting that, for both disorders, many medians for lost paid worktime (HALT questions 1 and 2) were zero, indicating that a minority (< 50%) of responders accounted for all the lost paid worktime reported.

**Table 1:** Headache-attributed disability by headache type and country, and migraine-attributed impairment by country (values are population means ± SEM [median])

Country	Migraine			Probable medication-overuse headache	
	pTIS (%)	Disability^**1**^ (%)	Impairment^**2**^ (arbitrary units)	pTIS (%)	Disability^**1**^ (%)
China	8.5 ± 0.7 [5.5]	3.8 ± 0.3 [2.4]	19.7 ± 1.6 [11.0]	–	–
Ethiopia	8.0 ± 0.3 [6.6]	3.5 ± 0.2 [2.9]	21.2 ± 0.9 [16.4]	61.6 ± 7.4 [66.7]	13.7 ± 1.6 [14.9]
India	5.9 ± 0.4 [2.7]	2.6 ± 0.2 [1.2]	15.1 ± 1.1 [6.6]	36.4 ± 5.9 [20.8]	8.1 ± 1.3 [4.7]
Lithuania + Luxembourg	8.9 ± 0.4 [5.5]	3.9 ± 0.2 [2.4]	20.1 ± 1.0 [11.2]	22.7 ± 3.0 [12.5]	5.1 ± 0.7 [2.8]
Nepal	5.9 ± 0.3 [3.3]	2.6 ± 0.1 [1.5]	14.2 ± 0.7 [6.8]	30.7 ± 4.1 [13.9]	6.9 ± 0.9 [3.1]
Pakistan	4.5 ± 0.2 [3.0]	2.0 ± 0.1 [1.3]	10.8 ± 0.5 [6.6]	49.9 ± 3.8 [37.5]	11.1 ± 0.8 [8.4]
Russia	7.1 ± 0.9 [3.7]	3.1 ± 0.4 [1.6]	17.2 ± 2.3 [8.2]	48.8 ± 4.6 [50.0]	10.9 ± 1.0 [11.2]
Spain	9.4 ± 0.5 [6.6]	4.1 ± 0.2 [2.9]	22.2 ± 1.3 [13.2]	24.3 ± 3.8 [10.4]	5.4 ± 0.9 [2.3]

pTIS: proportion of time in ictal state calculated at individual level as attack frequency*reported usual attack duration; ^1^ calculated at individual level as pTIS*disability weight from GBD2015 [49]; ^2^ calculated at individual level as pTIS*reported usual headache intensity.

**Fig. 1:**
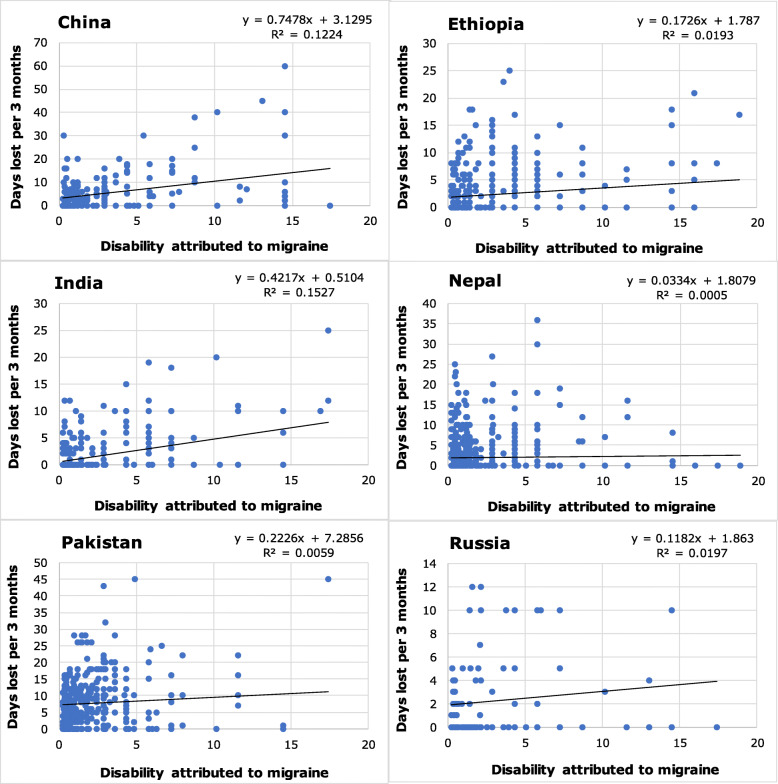
The relationships between disability attributed to migraine (calculated as proportion of time in ictal state*disability weight from GBD2015 [49]) and lost paid worktime (HALT questions 1 + 2) in the six countries with large and fully population-based samples. Values of R^2^ and β differ somewhat from those in Table 9 because of removal of extreme outliers that would otherwise compress the axes

**Table 2:** Lost productive time attributed to migraine by country (values are population means ± SEM [median])

Country	Lost productive time (days/3 months per person)				
	HALT question(s)^**1**^				
	**1**	**2**	**1 + 2**	**3 + 4**	**1 + 2 + 3 + 4**
China	2.4 ± 0.4 [0.0]	4.1 ± 0.5 [2.0]	6.5 ± 0.7 [3.0]	7.7 ± 0.8 [4.0]	14.0 ± 1.4 [8.0]
Ethiopia	1.2 ± 0.1 [0.0]	1.4 ± 0.1 [0.0]	2.7 ± 0.2 [0.0]	4.3 ± 0.4 [0.0]	6.2 ± 0.4 [4.0]
India	1.1 ± 0.1 [0.0]	0.6 ± 0.1 [0.0]	1.7 ± 0.2 [0.0]	2.8 ± 0.2 [1.0]	4.4 ± 0.3 [3.0]
Lithuania + Luxembourg	0.9 ± 0.3 [0.0]	1.9 ± 0.2 [0.0]	2.7 ± 0.3 [0.0]	4.5 ± 0.4 [2.0]	6.8 ± 0.5 [2.0]
Nepal	1.2 ± 0.1 [0.0]	0.8 ± 0.1 [0.0]	2.0 ± 0.2 [0.0]	3.6 ± 0.3 [1.0]	5.6 ± 0.4 [3.0]
Pakistan	3.3 ± 0.2 [2.0]	4.8 ± 0.3 [3.0]	8.1 ± 0.4 [6.0]	11.2 ± 0.4 [10.0]	13.9 ± 0.4 [12.0]
Russia	0.2 ± 0.1 [0.0]	2.0 ± 0.3 [0.0]	2.2 ± 0.3 [0.0]	3.9 ± 0.4 [3.0]	6.2 ± 0.7 [4.0]
Spain	1.4 ± 0.2 [0.0]	4.4 ± 0.3 [2.0]	5.6 ± 0.4 [3.0]	7.3 ± 0.5 [3.0]	12.3 ± 0.8 [6.0]

HALT: Headache-Attributed Lost Time questionnaire. ^1^ Questions 1 and 2 relate to work time (absenteeism and presenteeism respectively); questions 3 and 4 relate to household work (days with nothing or less than half of normal achieved) (see text).

**Table 3:** Lost productive time attributed to probable medication-overuse headache by country (values are population means ± SEM [median])

Country	Lost productive time (days/3 months per person)				
	HALT question(s)^**1**^				
	1	2	1 + 2	3 + 4	1 + 2 + 3 + 4
Ethiopia	8.9 ± 4.2 [0.0]	8.6 ± 3.7 [0.0]	17.4 ± 6.4 [0.0]	16.9 ± 5.1 [3.0]	32.8 ± 7.3 [20.0]
India	2.9 ± 2.0 [0.0]	1.2 ± 0.8 [0.0]	4.1 ± 2.3 [0.0]	9.9 ± 2.2 [7.0]	14.0 ± 3.5 [8.0]
Lithuania + Luxembourg	1.9 ± 0.8 [0.0]	4.5 ± 1.0 [0.0]	6.1 ± 1.4 [0.0]	18.9 ± 2.8 [11.5]	22.4 ± 2.9 [15.5]
Nepal	4.6 ± 1.4 [0.0]	2.9 ± 0.9 [0.0]	7.5 ± 2.1 [0.0]	9.9 ± 1.8 [5.0]	16.9 ± 3.0 [13.0]
Pakistan	3.4 ± 0.6 [0.0]	3.6 ± 0.6 [0.0]	6.9 ± 1.1 [1.0]	17.5 ± 1.7 [12.0]	23.0 ± 2.0 [18.0]
Russia	1.4 ± 0.7 [0.0]	3.8 ± 0.9 [0.0]	5.3 ± 1.3 [0.0]	12.6 ± 1.3 [11.5]	17.9 ± 2.0 [15.0]
Spain	4.4 ± 2.1 [0.0]	10.0 ± 1.7 [8.5]	13.4 ± 2.5 [10.0]	24.9 ± 3.3 [20.0]	34.7 ± 3.8 [28.0]

HALT: Headache-Attributed Lost Time questionnaire. ^1^ Questions 1 and 2 relate to work time (absenteeism and presenteeism respectively); questions 3 and 4 relate to household work (days with nothing or less than half of normal achieved) (see text).

**Fig. 2:**
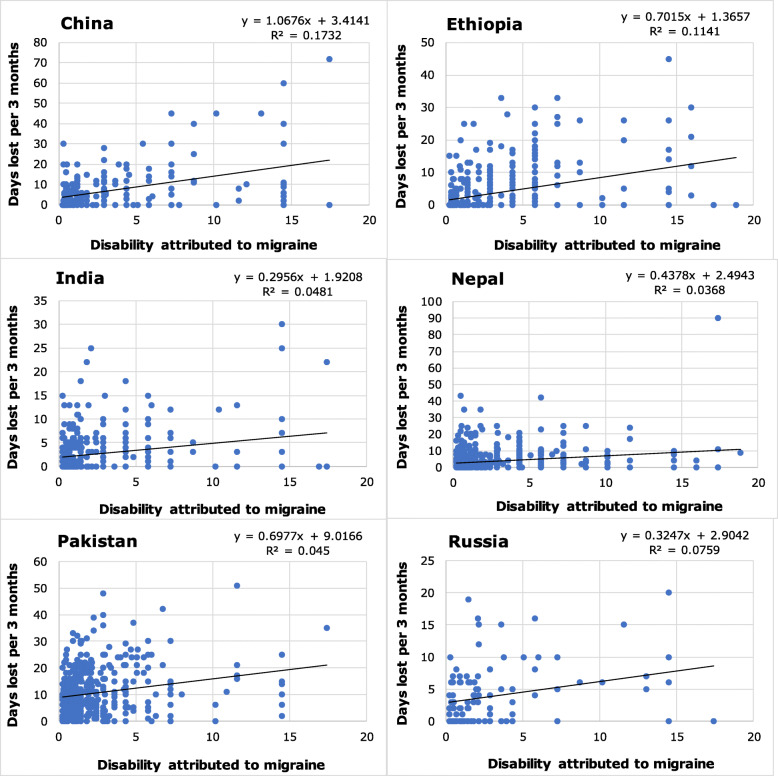
The relationships between disability attributed to migraine (calculated as proportion of time in ictal state*disability weight from GBD2015 [49]) and lost household worktime (HALT questions 3 + 4) in the six countries with large and fully population-based samples. Values of R^2^ and β differ somewhat from those in Table 11 because of removal of extreme outliers that would otherwise compress the axes

**Fig. 3:**
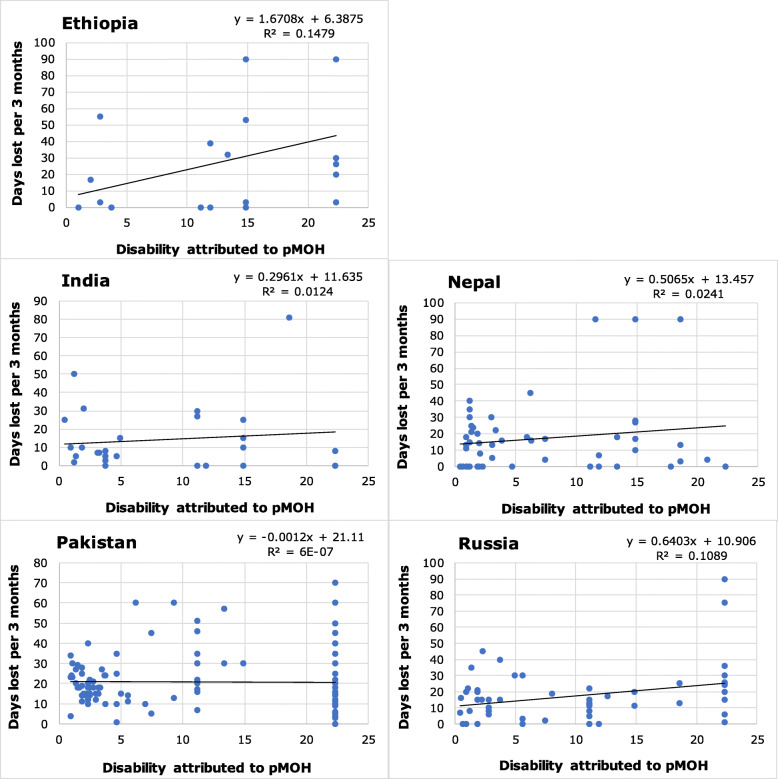
The relationships between disability attributed to probable medication-overuse headache (calculated as proportion of time in ictal state*disability weight from GBD2015 [49]) and total lost productivity (HALT questions 1 + 2 + 3 + 4) in adult population-based samples from five countries. Values of R^2^ and β differ somewhat from those in Table 9 because of removal of extreme outliers that would otherwise compress the axes

Spearman correlation analyses revealed variable and mostly weak correlations, occasionally negative, between disability and lost paid worktime for both headache types (Tables 4 and 5). Nonetheless, for total lost paid worktime attributed to migraine, correlations were significant in six of the nine countries and highly significant in four (r_s_ = − 0.07-0.30) (Table 4). Analyses of pMOH were inevitably affected by low numbers, but in Pakistan (*N* = 93) correlation was moderately and highly significantly negative (r_s_ = − 0.54) (Table 5). For lost household worktime, correlations were all positive for migraine, weak or moderate (r_s_ = 0.12–0.39), and significant or highly significant in all countries but India (Table 6). For pMOH, correlation was positive and significant only in Russia (Table 6). Correlations between disability and total lost productivity were always positive for migraine, weak or moderate (r_s_ = 0.11–0.37) but mostly highly significant, whereas for pMOH correlation was both positive and significant only in Russia (r_s_ = 0.32) and Spain (r_s_ = 0.28) (Table 7).

**Table 4:** Spearman correlations between disability^1^ and lost paid worktime attributed to migraine by country (values of N vary because of zero values in some responses to either question)

Country	HALT question 1 (absenteeism)			HALT question 2 (presenteeism)			HALT questions 1 + 2 (total lost paid worktime)		
	N	r_**s**_	p	N	r_**s**_	p	N	r_**s**_	p
China	199	0.10	0.18	200	0.17	**0.02**	200	0.18	**0.009**
Ethiopia	479	−0.05	0.32	479	−0.08	0.09	479	−0.07	0.14
India	372	0.17	**0.001**	372	0.16	**0.003**	372	0.24	**< 0.001**
Lithuania + Luxembourg	509	0.15	**< 0.001**	502	0.20	**< 0.001**	523	0.20	**< 0.001**
Nepal	674	0.02	0.59	674	0.01	0.88	674	0.00	0.93
Pakistan	394	0.05	0.34	400	0.11	**0.02**	418	0.13	**0.01**
Russia	108	−0.02	0.83	108	0.10	0.29	108	0.09	0.38
Spain	339	0.21	**< 0.001**	343	0.30	**< 0.001**	354	0.30	**< 0.001**

HALT: Headache-Attributed Lost Time questionnaire. ^1^ Calculated as proportion of time in ictal state*disability weight from GBD2015 [49]; significant *p*-values are emboldened.

**Table 5:** Spearman correlations between disability^1^ and lost paid worktime attributed to probable medication-overuse headache by country (values of N vary because of zero values in some responses to either question)

Country	HALT question 1 (absenteeism)			HALT question 2 (presenteeism)			HALT questions 1 + 2 (total lost paid work time)		
	N	r_**s**_	p	N	r_**s**_	p	N	r_**s**_	p
Ethiopia	23	0.15	0.49	23	0.08	0.72	23	0.20	0.37
India	27	0.05	0.82	27	0.27	0.17	27	0.11	0.58
Lithuania + Luxembourg	46	0.08	0.59	47	0.39	**0.01**	49	0.30	**0.04**
Nepal	53	0.05	0.73	53	0.08	0.59	53	0.04	0.80
Pakistan	93	−0.58	**< 0.001**	93	−0.51	**< 0.001**	94	−0.54	**< 0.001**
Russia	64	− 0.002	0.98	64	− 0.13	0.30	64	−0.8	0.52
Spain	45	0.05	0.73	46	0.18	0.22	49	0.21	0.15

HALT: Headache-Attributed Lost Time questionnaire. ^1^ Calculated as proportion of time in ictal state*disability weight from GBD2015 [49]; significant *p*-values are emboldened.

**Table 6:** Spearman correlations between headache-attributed disability^1^ and lost household worktime (HALT questions 3 + 4) by headache type and country

Country	Migraine			Probable medication-overuse headache		
	N	r_**s**_	p	N	r_**s**_	p
China	203	0.27	**< 0.001**	–	–	–
Ethiopia	479	0.28	**< 0.001**	23	0.05	0.82
India	372	0.05	0.30	27	−0.08	0.69
Lithuania + Luxembourg	543	0.30	**< 0.001**	58	−0.03	0.85
Nepal	674	0.12	**0.003**	53	0.17	0.22
Pakistan	483	0.23	**< 0.001**	104	−0.04	0.72
Russia	108	0.27	**0.005**	64	0.39	**0.002**
Spain	355	0.39	**< 0.001**	54	0.19	0.17

HALT: Headache-Attributed Lost Time questionnaire. ^1^ Calculated as proportion of time in ictal state*disability weight from GBD2015 [49]; significant *p*-values are emboldened.

**Table 7:** Spearman correlations between headache-attributed disability^1^ and total lost productivity (HALT questions 1 + 2 + 3 + 4) by headache type and country

Country	Migraine			Probable medication-overuse headache		
	N	r_**s**_	p	N	r_**s**_	p
China	203	0.28	**< 0.001**	–	–	–
Ethiopia	479	0.28	**< 0.001**	23	0.34	0.12
India	372	0.25	**< 0.001**	27	− 0.11	0.57
Lithuania + Luxembourg	554	0.30	**< 0.001**	62	0.08	0.52
Nepal	674	0.11	**0.004**	53	0.09	0.50
Pakistan	617	0.31	**< 0.001**	115	−0.14	0.14
Russia	108	0.28	**0.004**	64	0.32	**0.01**
Spain	367	0.37	**< 0.001**	55	0.28	**0.04**

HALT: Headache-Attributed Lost Time questionnaire. ^1^ Calculated as proportion of time in ictal state*disability weight from GBD2015 [49]; significant p-values are emboldened.

Spearman correlations between migraine-attributed impairment and lost paid worktime were again variable and mostly weak (r_s_ ≤ 0.34), negative (r_s_ = − 0.05) in Ethiopia, but significant or highly significant in six of the nine countries (Table 8). For lost household worktime, correlations were less variable, generally somewhat stronger (r_s_ = 0.18–0.44) and mostly highly significant, with India an outlier (r_s_ = 0.05). For total lost productivity, all correlations (r_s_ = 0.17–0.41) were highly significant except in Russia (Table 8).

**Table 8:** Spearman correlations between impairment^1^ and lost worktime attributed to migraine by country (values of N vary because of zero values in some responses to one or more questions)

Country	HALT question 1 + 2			HALT question 3 + 4			HALT questions 1 + 2 + 3 + 4		
	N	r_**s**_	p	N	r_**s**_	p	N	r_**s**_	p
China	200	0.22	**0.002**	203	0.30	**< 0.001**	203	0.32	**< 0.001**
Ethiopia	479	−0.05	0.27	479	0.31	**< 0.001**	479	0.32	**< 0.001**
India	372	0.28	**< 0.001**	372	0.05	0.30	372	0.28	**< 0.001**
Lithuania + Luxembourg	517	0.24	**< 0.001**	537	0.35	**< 0.001**	548	0.35	**< 0.001**
Nepal	674	0.01	0.73	674	0.18	**< 0.001**	674	0.17	**< 0.001**
Pakistan	416	0.14	**0.005**	481	0.24	**< 0.001**	613	0.32	**< 0.001**
Russia	108	0.07	0.49	108	0.25	**0.008**	108	0.25	**0.008**
Spain	351	0.34	**< 0.001**	352	0.44	**< 0.001**	364	0.41	**< 0.001**

HALT: Headache-Attributed Lost Time questionnaire. ^1^ Calculated as proportion of time in ictal state*reported usual headache intensity; significant p-values are emboldened.

Regression analyses told a similar story. For migraine-attributed disability and total lost paid worktime, values of R^2^ were in the range 0–0.13, yet associations in the linear model were highly significant in five of the nine countries (Table 9). For pMOH, with values of R^2^ in a similar range, only Pakistan showed significance (Table 10). For lost household worktime and total lost productivity, almost all associations were highly significant for migraine but values of R^2^ were still low (0.02–0.21) (Tables 11 and 12). For pMOH, only Russia showed significance. For migraine-attributed impairment, the picture was similar (Table 13).

**Table 9:** Linear regressions between headache-attributed disability^1^ and lost paid worktime attributed to migraine by country

Country	HALT question 1 (absenteeism)			HALT question 2 (presenteeism)			HALT questions 1 + 2 (total lost paid worktime)		
	R^**2**^	Equation	p	R^**2**^	Equation	p	R^**2**^	Equation	p
China	0.04	Y = 0.24x + 1.5	**0.003**	0.08	Y = 0.45x + 2.4	**< 0.001**	0.08	Y = 0.69x + 3.9	**< 0.001**
Ethiopia	0.00	Y = 0.06x + 1.0	0.15	0.01	Y = 0.08x + 1.2	0.07	0.01	Y = 0.14x + 2.2	0.07
India	0.08	Y = 0.24x + 0.4	**< 0.001**	0.07	Y = 0.17x + 0.2	**< 0.001**	0.12	Y = 0.41x + 0.6	**< 0.001**
Lithuania + Luxembourg	0.00	Y = 0.07x + 0.6	0.30	0.05	Y = 0.30x + 0.8	**< 0.001**	0.03	Y = 0.32x + 1.4	**< 0.001**
Nepal	0.00	Y = 0.00x + 1.2	0.94	0.00	Y = 0.02x + 0.8	0.57	0.00	Y = 0.02x + 2.0	0.76
Pakistan	0.00	Y = 0.08x + 3.2	0.34	0.01	Y = 0.16x + 4.6	0.11	0.01	Y = 0.21x + 7.4	0.15
Russia	0.00	Y = -0.01x + 0.2	0.72	0.03	Y = 0.13x + 1.6	0.09	0.02	Y = 0.12x + 1.9	0.15
Spain	0.09	Y = 0.25x + 0.3	**< 0.001**	0.12	Y = 0.50x + 2.4	**< 0.001**	0.13	Y = 0.69x + 2.8	**< 0.001**

HALT: Headache-Attributed Lost Time questionnaire. ^1^ Calculated as proportion of time in ictal state*disability weight from GBD2015 [49]; significant p-values are emboldened.

**Table 10:** Linear regressions between headache-attributed disability^1^ and lost paid worktime attributed to probable medication-overuse headache by country

Country	HALT question 1 (absenteeism)			HALT question 2 (presenteeism)			HALT questions 1 + 2 (total lost paid worktime)		
	R^**2**^	Equation	p	R^**2**^	Equation	p	R^**2**^	Equation	p
Ethiopia	0.06	Y = 0.60x + 0.7	0.28	0.00	Y = 0.01x + 8.4	0.98	0.02	Y = 0.61x + 9.1	0.48
India	0.00	Y = -0.09x + 3.6	0.77	0.15	Y = 0.23x – 0.6	0.05	0.01	Y = 0.14x + 3.0	0.70
Lithuania + Luxembourg	0.00	Y = -0.04x + 2.0	0.81	0.04	Y = 0.27x + 3.2	0.16	0.02	Y = 0.25x + 4.9	0.37
Nepal	0.02	Y = 0.23x + 3.1	0.28	0.03	Y = 0.16x + 1.8	0.23	0.03	Y = 0.39x + 4.9	0.23
Pakistan	0.08	Y = -0.18x + 5.7	**0.006**	0.15	Y = -0.25x + 7.0	**< 0.001**	0.13	Y = -0.43x + 12.5	**< 0.001**
Russia	0.04	Y = 0.14x – 0.05	0.11	0.00	Y = 0.03x + 3.6	0.81	0.02	Y = 0.16x + 3.5	0.31
Spain	0.01	Y = 0.19x + 3.6	0.64	0.04	Y = 0.47x + 8.0	0.19	0.03	Y = 0.53x + 11.1	0.28

HALT: Headache-Attributed Lost Time questionnaire. ^1^ Calculated as proportion of time in ictal state*disability weight from GBD2015 [49]; significant p-values are emboldened.

**Table 11:** Linear regressions between headache-attributed disability^1^ and lost household worktime (HALT questions 3 + 4) by headache type and country

Country	Migraine			Probable medication-overuse headache		
	R^**2**^	Equation	p	R^**2**^	Equation	p
China	0.15	Y = 1.04x + 3.8	**< 0.001**	–	–	–
Ethiopia	0.07	Y = 0.66x + 1.9	**< 0.001**	0.00	Y = 0.19x + 14.2	0.78
India	0.04	Y = 0.28x + 2.0	**< 0.001**	0.01	Y = 0.16x + 8.6	0.63
Lithuania + Luxembourg	0.12	Y = 0.73x + 1.7	**< 0.001**	0.01	Y = -0.28x + 20.4	0.60
Nepal	0.04	Y = 0.44x + 2.5	**< 0.001**	0.01	Y = 0.23x + 8.4	0.40
Pakistan	0.04	Y = 0.73x + 9.4	**< 0.001**	0.00	Y = -0.05x + 19.0	0.76
Russia	0.08	Y = 0.33x + 2.9	**0.004**	0.14	Y = 0.48x + 7.4	**0.002**
Spain	0.21	Y = 1.16x + 2.6	**< 0.001**	0.04	Y = 0.71x + 21.0	0.18

HALT: Headache-Attributed Lost Time questionnaire. ^1^ Calculated as proportion of time in ictal state*disability weight from GBD2015 [49]; significant p-values are emboldened.

**Table 12:** Linear regressions between headache-attributed disability^1^ and total lost productivity (HALT questions 1 + 2 + 3 + 4) by headache type and country

Country	Migraine			Probable medication-overuse headache		
	R^**2**^	Equation	p	R^**2**^	Equation	p
China	0.14	Y = 1.66x + 7.6	**< 0.001**	–	–	–
Ethiopia	0.07	Y = 0.79x + 4.1	**< 0.001**	0.06	Y = 1.04x + 18.5	0.28
India	0.13	Y = 0.69x + 2.7	**< 0.001**	0.01	Y = 0.30x + 11.6	0.58
Lithuania + Luxembourg	0.10	Y = 0.96x + 3.0	**< 0.001**	0.00	Y = 0.004x + 22.5	0.99
Nepal	0.02	Y = 0.46x + 4.4	**< 0.001**	0.02	Y = 0.51x + 13.5	0.27
Pakistan	0.06	Y = 1.11x + 11.5	**< 0.001**	0.00	Y = -0.10x + 23.3	0.56
Russia	0.07	Y = 0.44x + 4.8	**0.007**	0.11	Y = 0.64x + 10.9	**0.008**
Spain	0.18	Y = 1.69x + 5.3	**< 0.001**	0.04	Y = 0.89x + 30.0	0.14

HALT: Headache-Attributed Lost Time questionnaire. ^1^ Calculated as proportion of time in ictal state*disability weight from GBD2015 [49]; significant p-values are emboldened.

**Table 13:** Linear regressions between impairment^1^ and lost paid worktime, lost household worktime and total lost productivity attributed to migraine by country

Country	HALT question 1 + 2			HALT question 3 + 4			HALT questions 1 + 2 + 3 + 4		
	R^**2**^	Equation	p	R^**2**^	Equation	p	R^**2**^	Equation	p
China	0.11	Y = 0.15x + 3.4	**< 0.001**	0.13	Y = 0.18x + 4.1	**< 0.001**	0.14	Y = 0.32x + 7.6	**< 0.001**
Ethiopia	0.01	Y = 0.03x + 2.1	**0.03**	0.09	Y = 0.12x + 1.8	**< 0.001**	0.09	Y = 0.14x + 3.9	**< 0.001**
India	0.14	Y = 0.07x + 0.6	**< 0.001**	0.05	Y = 0.05x + 2.0	**< 0.001**	0.16	Y = 0.12x + 2.6	**< 0.001**
Lithuania + Luxembourg	0.03	Y = 0.06 + 1.4	**< 0.001**	0.13	Y = 0.14x + 1.7	**< 0.001**	0.12	Y = 0.19x + 3.0	**< 0.001**
Nepal	0.00	Y = 0.01x + 1.9	0.53	0.06	Y = 0.09x + 2.4	**< 0.001**	0.04	Y = 0.10x + 4.3	**< 0.001**
Pakistan	0.01	Y = 0.05x + 7.2	0.05	0.05	Y = 0.15x + 9.1	**< 0.001**	0.08	Y = 0.22x + 11.3	**< 0.001**
Russia	0.02	Y = 0.02x + 1.9	0.15	0.05	Y = 0.04x + 3.2	**0.03**	0.05	Y = 0.06x + 5.1	**0.03**
Spain	0.16	Y = 0.13x + 2.8	**< 0.001**	0.22	Y = 0.20x + 2.9	**< 0.001**	0.20	Y = 0.30x + 5.6	**< 0.001**

HALT: Headache-Attributed Lost Time questionnaire. ^1^ Calculated as proportion of time in ictal state*reported usual headache intensity; significant p-values are emboldened.

Illustrative scatter plots for the six countries with large and fully population-based samples are shown in Figs. 1, 2, 3, with linear trendlines and calculated values of β and R^2^. These values differ somewhat from those in the tables (which should be considered correct) because of removal of extreme outliers (see Methods). The figures are constructed to allow visual comparisons between countries. All show wide scatter, with the differences between countries highlighted by the scale differences on the Y axes. Evident in all, but more in some countries than in others, are numerous data points indicating high reported lost productivity despite low estimated disability.

Figures 1 and 2 show, respectively, lost paid and household worktime attributed to migraine. Disability values are the same in each, with a possible range of 0–22% based on DW = 0.441 [49] since maximum pTIS was 50% (cases of headache on ≥15 days/month were excluded). The theoretical maxima for lost productive time were 90 days for each, although lost paid worktime (Fig. 1) might be limited if related by the reporting participant to a 5- or 6-day working week. The degrees of scatter indicate complexity and involvement of other factors in the relationships.

Figure 3 shows total lost productivity attributed to pMOH. With maximum pTIS = 100%, the possible range for disability was also 0–22%, based on DW = 0.217 [49]. The theoretical maximum lost productive time was 90 days. Numbers were relatively low, but the degrees of scatter, particularly in Pakistan with negative β, again indicate complexity and involvement of other factors in the relationships. Also evident in these plots, especially in Pakistan and Russia, are the numbers who reported pTIS = 100%.

## Discussion

Our earlier systematic review established that the literature was silent on the relationship between headache-attributed disability and lost productivity [17]. This, therefore, is the first study to report it. We used individual-participant data from population-based studies in nine disparate countries, and considered the two headache types of greatest public-health importance from the point of view of causing disability. For migraine, in a linear model, we found positive associations with total lost paid worktime, significant in many of the countries and highly significant in some despite low values of R^2^ (0–0.16). With lost household worktime and total lost productivity (paid + household), associations were highly significant in almost all countries, still with quite low values of R^2^ (0.04–0.22). Analysing migraine-attributed impairment rather than disability made little difference. For pMOH, with relatively small numbers, associations were generally weaker, occasionally negative and mostly not significant.

All scatter plots revealed wide variations between individuals. Many with apparently high disability reported little or no lost productivity, while large numbers estimated to have little disability nonetheless reported substantial lost productivity. Within the ranges of variation, Spain, unique in including data obtained through a patient organisation [36], was often at the high extreme, though not an outlier.

Difficulties existed in measuring both disability and lost productivity [17, 50].

We defined “disability” in the sense used by GBD, expressing it as the product of pTIS and the DW from GBD2015 for the ictal state of the headache type in question [49]. Since DW was a constant for each headache type, this product reflected pTIS alone for the purpose of determining associations. Of the factors in the calculation of pTIS, frequency is a relatively objective count of days but the population-based enquiries depended on recall over representative periods (typically 3 months for frequencies up to 2–3/month, the common range). Duration assessment required not only similar recall, but also estimation of the average. Although attacks might be stereotyped for some people, this would not be true for all, and intervening sleep often obscures the beginnings or ends of attacks. Additionally, treatment effects, if any, might vary.

Introducing headache intensity into the product as a more nuanced reflection of symptom burden (“impairment”) might not have achieved this purpose for three reasons. First, it added to the subjectivity of the measure, since judgements of pain intensity are entirely subjective. Second, these judgements were insensitive, being rated in the original population-based enquiries on the usual verbal scale of “mild”, “moderate” or “severe”. This is not an interval scale but, by quantifying these as 1–3, again as usual, we treated it as such. Third, we doubt the independence of intensity and duration because more intense headache might be expected to have a longer resolution time.

On the dependent variable side, estimates of lost productivity were again based in the original enquiries on recall over the preceding 3 months, and were likely to be inexact. Further, whereas work-absence days and days of household work entirely lost might be clearly remembered, there was considerable subjectivity in judging productivity reduced to < 50% [48]. So-called presenteeism, accounting for the majority of lost work time [19, 22], has low visibility and is very difficult to measure [51].

In large populations, averages derived from large numbers sufficiently compensate for the generally non-systematic measurement errors arising from these factors. Population-based estimates of lost health and productivity are therefore reasonably sound. But it is a different matter at individual level. In correlation analyses based on IPD, all of these errors directly intrude, and will tend to disguise any associations.

So, too, will individual variation in behavioural response to impairment, the presumed driver of lost productivity – at least as estimated by HALT [48]. To the extent that people have a choice when they wake in the morning with headache, and with work beckoning, many external factors operate, influencing different people to very different degrees. “How ill do I feel?” may be determined directly by symptoms, but there are other response-determining questions that are personal and non-illness-related: “How important is my work?”, “How enjoyable is it?”, “Can I make up the time later?”, “Do other people depend on me for their own work?”, “Will I lose pay?”, even “Is the weather bad?” Coping mechanisms, developed as protection against the consequences of high-frequency headaches, also weigh in to maintain productivity that otherwise would be lost. Many of these factors would be less influential on household work, much of which is more flexible in its demands.

Other factors introduce variability between populations: cultural differences expressed in stoicism – in the reporting of pain and in responses to it; environmental influences; socioeconomic factors such as job insecurity limiting response options. In India, women report greater individual burden from headache than men [33], but local culture puts fewer women in paid work, reducing this potential element of lost productivity. For many in Ethiopia, poverty and dependence on production mandate that work continues whatever the adversity [15]. The range of other determinants of lost productivity, apart from disability, is not known but may vary with culture, geography, climate, population wealth and living standards, and general health [14]. While it was not a purpose of this study to make comparisons between countries, the differences that came to light in our analyses were probably manifestations of all of these.

For all of these reasons, the individual variability revealed by the scatter plots is unsurprising. But there are also two disease-related factors that might have been in play. First, it was reported in one of the population studies that lost productivity due to migraine exceeded disability expected from time spent with headache [33], suggesting that the disabling effect of migraine outlasted headache. This was not interictal burden, reported by many people with migraine [29]: the study in India described motivation and energy lost during migraine as ictal symptoms that might for some time outlast the headache phase of attacks [33]. These might not be captured in pTIS, but still impair productivity. Interictal burden, on the other hand, which was ignored in our analyses (and is ignored in the GBD studies), would not be factored into disability estimates based on pTIS, but neither would we expect it to have an effect on lost productivity. Second, it would not be unexpected if occasional, infrequent attacks, being less predictable, were relatively more disruptive to productivity; if coping mechanisms invoked with more frequent episodes tempered this effect; and if these tended to become overwhelmed at high frequencies. These influences might lead to a more sigmoid relationship.

High significance in a linear model coupled with low R^2^ is not uncommon as a statistical phenomenon [52]. R^2^ is often misinterpreted as a goodness-of-fit measure but, more correctly, it is a reflection of the scatter of data points around the fitted regression line: a wide scatter due to high variance leads to low R^2^ [52]. When R^2^ values are low but the regression coefficients (β) are statistically significant, conclusions can still be drawn about the relationship between independent and dependent variables on a population level since the coefficients represent the average change in the latter given one unit change in the former [53]. But, since disability and lost productivity do not have common units, interpretation of β involves corrections of scale. In Tables 14 and 15, we work through the interpretation for all nine countries (including the three – Russia, Luxembourg and Spain – for which our earlier economic analyses were performed [54.55]), ordered by increasing population-mean disability (Table 14) or impairment (Table 15).

**Table 14:** Interpretation of regressions between total lost productivity (LP) and migraine-attributed disability (maD) in all countries ordered by increasing population mean maD

Value item	Country							
	Pakistan	Nepal	India	Russia	Ethiopia	China	Lithuania + Luxembourg	Spain
World Bank income ranking [54]	lower middle	lower middle	lower middle	upper middle	low	upper middle	high	high
Population mean maD^1^ (%)	2.0	2.6	2.6	3.1	3.5	3.8	3.9	4.1
Regression equation^2^ (Y=)	1.11x + 11.5	0.46x + 4.4	0.69x + 2.7	0.44x + 4.8	0.79x + 4.1	1.66x + 7.6	0.96x + 3.0	1.69x + 5.3
Population-level LP^3^ (Y):								
Y_1_ where x = maD	13.72	5.60	4.49	6.16	6.87	13.91	6.74	12.23
Y_2_ where x = 0.5*maD^4^	12.61	5.00	3.60	5.48	5.48	10.75	4.87	8.76
Y_2_/Y_1_	0.92	0.89	0.80	0.89	0.80	0.77	0.72	0.72
Recovery of LP for 50% reduction in maD (%)^5^	8	11	20	11	20	23	28	28
Pro rata recovery of LP per unit reduction in maD	0.16	0.22	0.40	0.22	0.40	0.46	0.56	0.56

^1^From Table 1; ^2^ from Table 12; ^3^ calculated from regression equation; ^4^ assuming intervention has achieved 50% reduction; ^5^ calculated as [1-(Y_2_/Y_1_)]*100; see text for further explanations

**Table 15:** Interpretation of regressions between total lost productivity (LP) and migraine-attributed impairment (maI) in all countries ordered by increasing population mean maI

Value item	Country							
	Pakistan	Nepal	India	Russia	China	Lithuania + Luxembourg	Ethiopia	Spain
Population mean maI^1^ (arbitrary units)	10.8	14.2	15.1	17.2	19.7	20.1	21.2	22.2
Regression equation^2^ (Y=)	0.05x + 7.2	0.01x + 1.9	0.07x + 0.6	0.02x + 1.9	0.15x + 3.4	0.06 + 1.4	0.03x + 2.1	0.13x + 2.8
Population-level LP^3^ (Y):								
Y_1_ where x = maI	7.74	2.04	1.66	2.24	6.36	2.60	2.74	5.69
Y_2_ where x = 0.5*maI^4^	7.47	1.97	1.13	2.07	4.88	2.00	2.42	4.24
Y_2_/Y_1_	0.97	0.97	0.68	0.93	0.77	0.77	0.88	0.75
Recovery of LP for 50% reduction in maI (%)^5^	3	3	32	7	23	23	12	25
Pro rata recovery of LP per unit reduction in maI	0.06	0.06	0.64	0.14	0.46	0.46	0.24	0.50

^1^From Table 1; ^2^ from Table 13; ^3^ calculated from regression equation; ^4^ assuming intervention has achieved 50% reduction; ^5^ calculated as [1-(Y_2_/Y_1_)]*100; see text for further explanations

For each country, Table 14 applies the regression equation from Table 12 first to the population mean migraine-attributed disability (maD) from Table 1 (calculating Y_1_) then to 0.5*maD (calculating Y_2_), assuming a hypothetical 50% reduction achieved through intervention. The equation {[1-(Y_2_/Y_1_)]*100} gives the percentage recovery of lost productivity expected from 50% reduction in population disability. The last row calculates the pro rata recovery of lost productivity per unit reduction in population disability, in the range 16–56%. Table 15 follows the same steps for impairment, with pro rata recovery of lost productivity per unit reduction in the range 6–64%.

We did not attempt similar interpretations for pMOH-attributed disability since most regressions were not significant.

### Implications for economic analyses

Headache disorders – particularly migraine and MOH – have very large detrimental effects on public health and production [1–10, 55]. Health politicians should wish to address these, but with regard to cost-effectiveness. Efficacious [46] and cost-effective treatments exist [56–58], but substantial investment in health care is needed to deliver these equitably, and with optimum efficiency, to all who need them [18]. These investments will be offset in proportion to whatever part of lost productivity is recovered through improved care [56, 57], an important consideration since the indirect costs of lost productivity are by far the larger proportion of all costs of headache [12–16, 19–24].

In our earlier economic analyses from the societal perspective (including indirect costs), investment in structured headache services [18] would be *cost saving*, not merely cost-effective, for all headache types, across health-care systems and in both 1-year and 5-year time frames, if mitigating disability recovered, proportionately, only 20% of lost productivity [57]. Our findings here indicate that, in many but not all countries, this will be comfortably achieved for migraine. Recovered proportion appears to be unrelated to country income level (Table 14), and Pakistan is a clear exception. We cannot say the same for MOH – which is not to say that it will not be, only that we could not show it with the much smaller samples.

We should note, however, that for both disorders the relationship between disability and lost productivity is complicated and weakened by a welter of interfering external factors, with high variance reflected at *population level* in low values of R^2^. At *individual level*, and in the context of treatment this is the level that matters, these external factors are mostly constant, or at least much less variable, so that much higher levels of correlation might be expected. The empirical evidence for this, however, has not been adduced, and is unlikely to be: it would require longitudinal interventional studies that are not only challenging to perform but also at high risk of outcomes influenced by the knowledge of being observed (the Hawthorne effect [59]).

### Strengths and limitations

Study strengths include the utilisation of IPD from nine studies conducted in very disparate countries, most with large and population-based samples [14–16, 33–35]. The particular value of the LTB studies lies in their similar methodology [38, 39], with multi-stage cluster sampling procedures ensuring samples demographically matched to their respective country populations*.* The study included the two headache disorders of greatest public-health importance.

As for limitations, those inherent in data dependent on subjective evaluation and recall appear unavoidable. LTB studies have introduced enquiry into headache yesterday [39] to obviate recall error in assessments of both disability and lost productivity, but the numbers, for now, remain small (1-day prevalence of migraine is low). Furthermore, the 1-day timeframe offers limited scope for variation in pTIS. Limitations specific to this study are that we chose not to include TTH (since this has relatively low priority for intervention, and most cases do not require professional care) and the low numbers with pMOH despite large samples.

## Conclusion

We achieved our purpose of examining and characterising, for the first time, the relationship between headache-attributed disability and lost productivity. We found significance or high significance in a linear regression model. Relief of disability through effective treatment of migraine can be expected, in most countries, to recover, pro rata, > 20% of lost productivity, with country income level not a factor. While any recovery will offset costs of care provision, our earlier analyses indicate that investment in structured headache services will be cost saving, not merely cost-effective, if proportionate recovery of lost productivity is above this level. This is, therefore, a very important finding for health policy, greatly strengthening the economic argument for this form of intervention. Furthermore, it is likely that a stronger relationship exists at individual level, where many of the extraneous factors are constant.

Introducing headache intensity into our analyses, attempting to reflect impairment and a more nuanced assessment of individual health loss, was not useful for reasons explained. We wonder whether analyses based on frequency alone as the independent variable, eliminating the subjectivity of intensity estimates and the uncertainties of duration, would show stronger association. This is something for future studies: the data exist.

## Data Availability

Not applicable.

## Abbreviations

DW: disability weight
GBD: Global Burden of Disease (study)
HALT: headache-attributed lost time (indices)
HARDSHIP: Headache-Attributed Restriction, Disability, Social Handicap and Impaired Participation (questionnaire)
ICHD: International Classification of Headache Disorders
IPD: individual-participant data
LTB: *Lifting The Burden*
maD: migraine-attributed disability
maI: migraine-attributed impairment
MOH: medication-overuse headache
pTIS: proportion of time in ictal state
SD: standard deviation
SEM: standard error (of mean)
TIS: time in ictal state
TTH: tension-type headache
WHO: World Health Organization
YLD: year lived with disability
